# One-Pot Synthesis of MnO_x_-SiO_2_ Porous Composites as Nanozymes with ROS-Scavenging Properties

**DOI:** 10.3390/nano12193503

**Published:** 2022-10-07

**Authors:** M. Dolores Garrido, Jamal El Haskouri, María D. Marcos, Francisco Pérez-Pla, José Vicente Ros-Lis, Pedro Amorós

**Affiliations:** 1 Institut de Ciència dels Materials (ICMUV), Universitat de València, Catedrático José Beltrán 2, 46980 Paterna, Spain; 2 Centro de Reconocimiento Molecular y Desarrollo Tecnológico (IDM), Unidad Mixta Universitat Politècnica de Valencia, Universitat de València, Departamento de Química, Universitat Politècnica de Valencia, 46022 Valencia, Spain; 3 CIBER de Bioingenieria, Biomateriales y Nanomedicina (CIBER-BBN), 28029 Madrid, Spain; 4 Centro de Reconocimiento Molecular y Desarrollo Tecnológico (IDM), Unidad Mixta Universitat Politècnica de Valencia, Universitat de València, Departamento de Química Inorgánica, Universitat de València, Doctor Moliner 56, 46100 Valencia, Spain

**Keywords:** nanozyme, manganese oxides, silica, mesoporous, catalase, superoxide dismutase

## Abstract

The development of nanomaterials that mimic the activity of enzymes is a topic of interest, for the decomposition of reactive oxygen species (ROS). We report the preparation of a novel nanocomposite of MnO_x_ needles covered with SiO_2_ porous material. The material was prepared in one pot with a two-step procedure. The material was characterized by EDX, SEM, TEM, XRD, nitrogen adsorption–desorption isotherms, and XPS. The synthesis protocol took advantage of the atrane method, favoring the nucleation and initial growth of manganese oxide needles that remained embedded and homogeneously dispersed in a mesoporous silica matrix. The final composite had a high concentration of Mn (Si/Mn molar ratio of ca. 1). The nanozyme presented bimodal porosity: intraparticle and interparticle association with the surfactant micelles and the gaps between silica particles and MnO_x_ needles, respectively. The porosity favored the migration of the reagent to the surface of the catalytic MnO_x_. The nanozyme showed very efficient SOD and catalase activities, thus improving other materials previously described. The kinetics were studied in detail, and the reaction mechanisms were proposed. It was shown that silica does not play an innocent role in the case of catalase activity, increasing the reaction rate.

## 1. Introduction

Enzymes are mainly protein-based biological catalysts that are responsible for controlling many physiological processes. All the reactions that involve enzymes occur with high catalytic activity and high specific activity under very specific conditions [1,2]. However, typical inorganic catalysts have the advantage that they can be used under extreme conditions, such as high temperature, high pressure, and extreme pH. If natural enzymes are exposed to these conditions, they denaturalize easily due to the incompatibility of natural enzymes out of physiological conditions. Several factors limit the practical application of natural enzymes for industrial uses due to high cost of preparation, low operational stability, and difficulties in recycling and reusing [3].

Enzyme mimetic compounds are low-molecular-weight complexes that present catalytic properties similar to natural enzymes, but they are sensitive to some environmental conditions and also present less activity and selectivity than metalloenzymes [4]. Just as natural enzymes have a protein backbone that aids in the catalytic process, enzyme mimetic compounds are surrounded by flexible ligands that simulate the environment of the metal center in the metalloenzyme [4,5]. There are complexes with different metal centers, such as Ni, Cu, Fe, Mn, Pt, and Zn, among others [4,5,6,7,8,9,10,11]. Many of these complexes have poor solubility in water and can suffer hydrolysis reactions or metal dissociation and oligomerization; to avoid these problems, a catalyst can be immobilized in mesoporous silica support to improve its stability [12,13].

The term nanozyme refers to nanomaterials with enzymatic catalytic properties that present many advantages, such as thermal stability, multifunctionality, ease of mass production, stable structure, high stability, low cost, and robustness to harsh environments [14,15]. For all these reasons, nanozymes are a good candidate for substituting natural enzymes in various applications since they can catalyze the same substrates as natural enzymes [16]. Nanozymes can be divided into two groups [3,14]: (a) enzymatic catalytic groups or enzymes that are anchored in nanomaterials, called nanomaterial hybrid enzymes. In this case, the main function of the nanomaterial is to improve the stability and durability of the anchored enzymes [17]. The enzyme can be immobilized through physical adsorption in which the enzyme is weakly bonded to the material or covalently bonded where the enzyme has been chemically modified [18]. (b) The nanomaterial is the one that possesses the catalytic activity without the need for anchored enzymes on its surface. On this occasion, the mechanism used in the catalytic process is like the natural enzyme.

Nanomaterials mimic some kinds of enzymes, such as peroxidase, oxidase, catalase, and superoxide dismutase (SOD) [19,20]. For example, ferromagnetic nanoparticles (Fe_3_O_4_) have been extensively studied due to their peroxidase-like activity and can catalyze substrate oxidation in the presence of H_2_O_2_ [21,22,23]. Other nanomaterials, such as noble metal nanoparticles, exhibit oxidase activity, and they can catalyze substrate oxidation with the assistance of O_2_ [24,25,26]. One of the most studied materials that presents catalase and dismutase activity is ceria (CeO_2_). This material can catalyze the reduction of H_2_O_2_ to water (catalase-like activity) and also can catalyze the dismutation of ^·^O_2_^-^ radicals to H_2_O_2_ (superoxide-dismutase-like activity) [27,28,29,30,31,32].

On the other hand, manganese oxides (MnO_x_) have been recently proposed as a nanozyme because they present several enzyme-like activities and are biocompatible and biodegradable [33,34,35]. To increase the biocompatibility of these nanoparticles, they can be covered with BSA (bovine serum albumin) [12,36]. In addition, their dispersibility and stability can be increased by PEG (polyethylene glycol) functionalization [34]. Furthermore, they can be loaded into a mesoporous matrix [35,37] or in polymer capsules [38], providing a more stable alternative to the traditional encapsulation of natural enzymes. MnO_x_ can produce ROS spontaneously under physiological conditions [39] or can scavenge ROS [33,40]. In order to eliminate reactive oxygen species, manganese oxides mimic different natural enzymes, such as catalase, peroxidase, superoxide dismutase, oxidase, and glutathione peroxidase. As MnO_x_ can mimic different natural enzymes at the same time, they are useful to use under high-oxidative-stress conditions [33,34,35,36,37,38,39,40,41,42]. The manganese oxide most studied has been MnO_2_ [33,35,37,38,39,40,41], but there have been some works on the enzyme-mimicking capability of M_2_O_3_ [36] and Mn_3_O_4_ [34,36,42].

Among the synthesis procedures of oxides, the atrane method allows balancing the hydrolysis and condensation processes of silicon and different elements through the formation of complexes containing triethanolamine-derived ligands (atrane complexes) [43]. Usually, this method leads to a high heteroelement dispersion along the silica walls, avoiding undesired phase segregation phenomena. This preparative route was discovered by our group and has been successfully applied for the isolation of a large variety of inorganically functionalized materials based on MCM-41 silicas [44,45,46,47,48,49], hierarchical mesoporous solids (UVM-7 type) [50,51,52,53,54,55], xerogels (UVM-11) [56], layered silicas [57], and phosphates [58].

We hypothesize that the atrane route can be a proper approach for the synthesis of a composite with ROS-scavenging properties based on manganese oxide particles dispersed homogeneously in a porous silica matrix to avoid the aggregation of the manganese active domains. Although the MnO_x_ are covered with silica, they may be accessible to the medium due to the porous structure that presents the coating. The resulting composite possesses catalase-like and superoxide-dismutase-like activities. Thus, the composite can be used to catalyze the elimination of H_2_O_2_ and can be used to catalyze the dismutation of ^˙^O_2_^-^ into H_2_O_2_.

## 2. Experimental Methods

### 2.1. Reagents and Materials

All the synthesis reagents were analytically pure and were used as received from Aldrich, including MnCl_2_, 2,2′,2″-nitrilotriethanol or triethanolamine (N(CH_2_-CH_2_-OH)_3_, hereinafter TEAH3), tetraethyl orthosilicate (TEOS), cetyltrimethylammonium bromide (CTAB), ethanol, hydrogen peroxide, sulfuric acid, potassium permanganate, phosphate-buffered saline (PBS), L-methionine, nitrotetrazolium blue chloride (NBT), (-)-riboflavin, and methylviologen.

### 2.2. Synthesis

**Figure 1:**
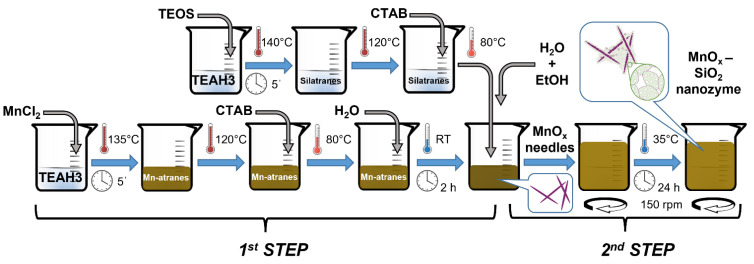
Schematic procedure of the “one-pot” two-step synthesis. The first stage involved, on the one hand, the independent preparation of the respective atrane complexes of silicon and manganese, as well as the hydrolysis and subsequent condensation of the Mn species (for a time of 2 h). The second stage involved the incorporation of the silatrane complexes that hydrolyzed and condensed on the MnO_x_ needles, which acted as seeds on which the formation of mesoporous silica occurred.

The synthesis strategy could be considered a “one-pot” two-step protocol (Figure 1) to stabilize the two main components that were finally mixed to form the nanozyme, or nanocomposite. First, 1.4 g of MnCl_2_ were dissolved in 10 mL of TEAH3 and heated at 135 °C for 5 min to form the Mn-atrane complexes. The resulting brown solution was cooled to 120 °C and 50 mg of CTAB was added while stirring. When the temperature dropped to 80 °C, 100 mL of water were added and kept under stirring for 2 h at 150 rpm (Suspension 1). On the other hand, 11 mL of TEOS and 23 mL of TEAH3 were mixed and heated at 140 °C for 5 min to form the silatrane complexes. The resulting solution was cooled to 120 °C and 4.5 g of CTAB was added while stirring. When the temperature dropped to 80 °C, Suspension 1 was added, followed by 350 mL of water and then 250 mL of ethanol. The mixture was left to age for 24 h at 35 °C. The resulting mesostructured brown powder was filtered off, washed with ethanol, and heated at 80 °C for 24 h. Finally, to open the mesoporous system, the surfactant was removed from the synthesized solid by calcination, increasing the temperature at a rate of 3 °C/min until reaching 550 °C and maintaining this temperature for 6 h in a static-air atmosphere, obtaining a dark brown powder.

### 2.3. Materials Characterization

The Mn and Si contents were determined with energy-dispersive X-ray spectroscopy (EDX) analysis using a scanning electron microscope (Philips-SEM-XL 30). The Si/Mn molar ratio values averaged from EDX data corresponding to ca. 25 different particles of each sample are summarized in Table 1. Powder X-ray diffraction (XRD) was carried out using a Bruker D8 Advance diffractometer with a monochromatic Cu Kα source operated at 40 kV and 40 mA. Patterns were collected in steps of 0.02° (2θ) over the angular range of 1–10.0° (2θ) with an acquisition time of 25 s per step. Additionally, XRD patterns were recorded over a wider angular range, 10–80° (2 θ), to determine the presence of segregated crystalline phases. For TEM, the samples were dispersed in ethanol, placed onto a carbon-coated copper microgrid, and left to dry before observation. TEM and STEM images were acquired with a JEOL 2100F microscope operated at 200 kV. Nitrogen adsorption–desorption isotherms were recorded with an automated Micromeritics ASAP2020 instrument. Before the adsorption measurements, the samples were outgassed in situ in a vacuum (10^−6^ Torr) at 110 °C for 15 h to remove adsorbed gases. X-ray photoelectron spectroscopy (XPS) measurements were collected with a SPECS spectrometer equipped with a Phoibos 150 MCD 9 analyzer and using a nonmonochromatic Al Kα (1486.6 eV) X-ray source. Spectra were recorded at 25 °C using an analyzer pass energy of 30 eV, an X-ray power of 50 W, and under an operating pressure of 10^−9^ mbar. During data processing, binding energy (BE) values were referenced to the C 1s peak settled at 284.5 eV. Samples were prepared by sticking, without sieving, the nanozyme onto a molybdenum plate with scotch tape film, followed by air drying.

### 2.4. Determination of the Catalase Activity

The catalase activity was determined by a titration of water peroxide with potassium permanganate. If the material presented catalase activity, the decomposition of water peroxide took place.

In a typical experiment, a certain amount of the Mn nanozyme was suspended in a solution of 1% water peroxide under stirring at 25 °C for 15 min. After this time, 1 mL of the dispersion was extracted and added to 50 mL of 0.1 M sulfuric acid for titration with 0.02 M potassium permanganate. In another experiment, 20 mg of the composite was suspended in 10 mL of water, and 10 mL of 2% water peroxide was added to the composite dispersion under stirring at 25 °C. At a given time, 1 mL of the dispersion was extracted and added to 50 mL of 0.1 M sulfuric acid for titration with 0.02 M potassium permanganate. Different kinetic experiments were conducted to determine the reaction mechanism, such as changing the concentration of water peroxide or introducing methylviologen into the reaction medium. In addition, to determine the importance of the silica in the reaction mechanism, we conducted a study of the decomposition of water peroxide with a material that was not covered with silica. The hydrogen peroxide elimination was calculated using the following Equation (1):

(1) $$H_{2}O_{2}\;\mathrm{Elimination}\left(\%\right)=\frac{{\left[H_{2}O_{2}\right]}_{0}-{\left[H_{2}O_{2}\right]}_{t}}{{\left[H_{2}O_{2}\right]}_{t}}\cdot 100$$

### 2.5. Determination of the Superoxide Dismutase Activity

The superoxide dismutase activity (SOD activity) of the composite was determined using a photoreduction method of NBT in a buffer solution (PBS, pH = 7.4). When riboflavin is irradiated with light in the presence of methionine, it reacts with oxygen to produce superoxide radicals. These superoxide radicals reduce nitrotetrazolium blue chloride to formazan, a blue-colored product with a maximum absorption at 560 nm. Therefore, if the composite presented SOD activity, the reduction of NBT was inhibited.

In a typical procedure, a certain amount of the composite was suspended in 8 mL of PBS containing 4.62·10^−5^ M of NBT, 0.01 M of methionine, and 3.56·10^−6^ M of riboflavin. The absorbances at 560 nm for each sample before (A_bef_) and after (A_aft_) being irradiated (10 min with a UV light (50 W)) were measured using a UV-vis spectrometer (Jasco V-770). Background absorbance was corrected by subtracting the value of the unilluminated mixture (A_bef_) from the illuminated mixture (A_aft_). The percentage inhibition rate was calculated using the following Equation (2):

(2) $$\mathrm{Inhibition}\;\mathrm{Rate}\left(\%\right)=\frac{A_{\mathrm{blank}}-A_{\mathrm{sample}}}{A_{\mathrm{blank}}}\cdot 100$$

## 3. Results and Discussion

### 3.1. Synthesis Strategy

The synthesis strategy employed made use of atrane complexes as true hydrolytic precursors [43]. In this sense, we could consider our synthesis protocol encompassed within the atrane method. As expected, if the hydrolysis and condensation processes of the Si and Mn atrane complexes started simultaneously, porous materials with good dispersion of Mn along the pore walls were achieved, avoiding the segregation of the Mn-rich oxidic domain [55]. However, in this case, our objective was to favor the formation of MnO_x_ domains to act as nanozymes. For this reason, we did not carry out a typical “one-pot/one-step” synthesis and provided some advantage to manganese to favor the formation of MnO_x_ nanocrystals simply by initiating the hydrolysis and condensation of the Mn-atrane complexes earlier in the absence of Si reagents. In a previous work, we determined through FAB that the dominant Mn-atrane complexes in the reaction medium before the addition of water were Mn(TEA)H_2_^+^, Mn(TEA)H_3_Cl^+^, Mn(TEA)_2_H_5_^+^, Mn_2_(TEA)_2_H_3_^+^, and Mn_3_(TEA)_3_H_4_^+^ [55]. These complexes (or mixtures of them) containing ligands derived from triethanolamine were our true hydrolytic precursors. Once the hydroalcoholic mixture was added, the hydrolysis and condensation began, which induced the appearance of MnO_x_ nuclei and their subsequent growth. The hydrolysis of all these complexes was expected to be initiated by the coordination positions most favorable to attack by ligands such as H_2_O or OH species; these were never those of the atrane entities [43,59]. Therefore, when triethanolamine acted, at least as a tridentate ligand, it exerted a protective effect with respect to metal hydrolysis (slowing down the hydrolysis rate), regardless of the mono-, bi-, or trinuclear nature of the starting complexes. After 2 h of reaction, a brown suspension was observed that could be associated with the nucleation and growth of manganese oxide crystals. Silatranes (also as a mixture of different complexes: Si(TEA)_2_H_2_, Si_2_(TEA)_3_H, and Si_3_(TEA)_4_) [43,60] were then added. To avoid complete coverage of the MnO_x_ domains (which would mean a significant loss in catalytic activity), CTAB was added to generate the formation of mesopores and, consequently, the accessibility of the different substrates to the active centers of Mn was preserved. It should be noted that the addition of silatranes and CTAB (in hydroalcoholic medium) generated a rapid turbidity in the reaction medium, faster than that in the absence of the MnO_x_ nanocrystal suspension. This could perhaps be due to the role that MnO_x_ crystals could play as seeds in the mesoporous silica’s subsequent precipitation process. Then, the formation of the second counterpart of the composite, the mesoporous silica, played a double role, preventing the aggregation of MnO_x_ crystals while maintaining their accessibility.

### 3.2. Nanozyme Characterization

Although we are aware that our material is really a composite with segregation (really wanted) of Mn-rich species and others that are fundamentally siliceous, we used EDX to assess the stoichiometry and chemical homogeneity of the final composite at the micrometer scale (using a 1 µm^3^ spot area). The Si/Mn molar ratio value was determined from data corresponding to approx. 25 independent measurements on different particles. By facilitating the formation of MnO_x_ domains in the first stage during the synthesis and also due to the greater solubility of the silica species [61], the final Mn content was relatively high for a heterogeneous silica material [55]: Si/Mn molar ratio = 1.01 ± 0.06. The small estimated standard deviation was consistent with the high chemical homogeneity of the composite at the microscale.

**Figure 2:**
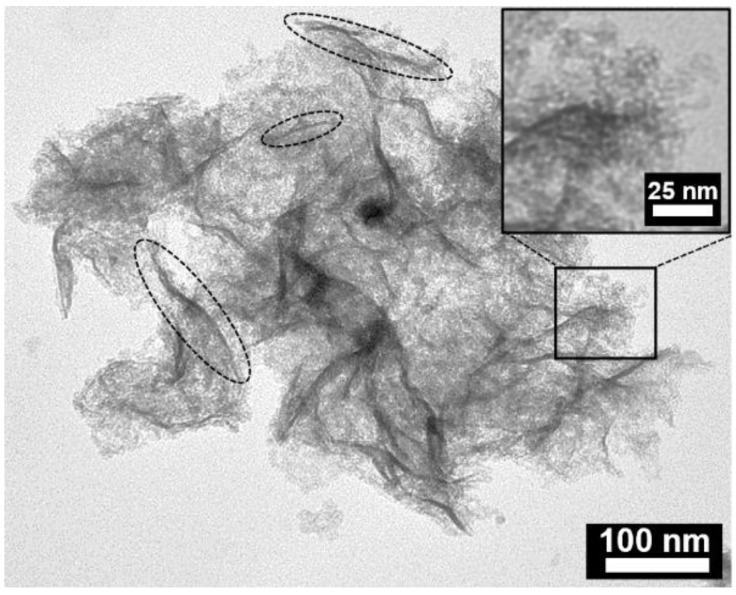
TEM images of the Mn-nanozyme. Some MnO_x_ needles are marked. In the inset, an enlarged image is observed in which both the mesoporous nature of the Si-rich nanoparticles and their small size can be seen.

TEM images provided a clear view of the morphology and organization of the composite (Figure 2). Darker needle-shaped domains were observed that corresponded to the MnO_x_ crystals that were generated during the first reaction stage (in the absence of Si reagents). These needles presented a marked anisotropy, with means lengths that oscillated in a wide range (100 ± 60 nm) but never exceeded 200 nm. On the contrary, they showed a reasonable homogeneity of sizes in the perpendicular axis (diameters around 10–15 nm). It was also observed that the formation of mesoporous silica prevented the Mn oxide needles from agglomerating. Mesoporous silica grew around the needles (which probably acted as crystallization seeds) and had a morphology reminiscent of UVM-7 type silica [62,63,64,65]: aggregates of mesoporous silica nanoparticles that generate a second (larger) pore system between particles and in whose formation MnO_x_ needles also contribute. The mesoporous silica nanoparticles were very small, with sizes in the 10–15 nm range. This size, significantly smaller than that observed in UVM-7 type silicas (ca. 30–40 nm), could be related to a very fast nanoparticle nucleation process (on the seeds), consuming a large part of the reagents and limiting their subsequent growth step. In fact, in some silica nanoparticles an extremely low number of mesopores could be observed (in the form of lighter spots): <6–10 mesopores per nanoparticle.

**Figure 3:**
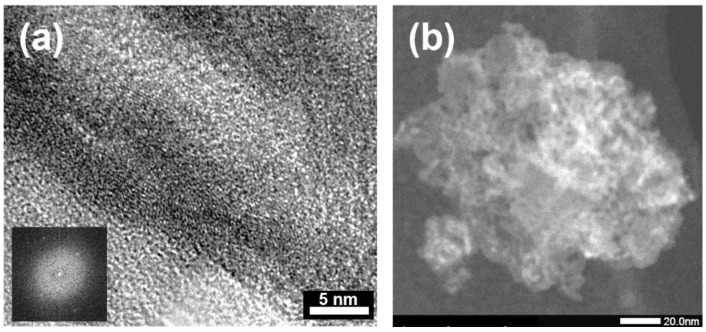
(**a**) HRTEM image of the Mn-nanozyme. The inset shows the presence of diffraction spots that confirm the crystalline nature of the MnO_x_ needles; (**b**) STEM-HAADF images of the Mn-nanozyme.

**Figure 4:**
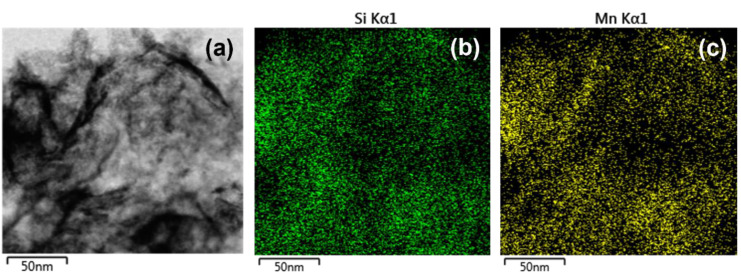
STEM image (**a**) and mapping showing the Si (**b**) and Mn (**c**) distributions along the nanozyme.

HRTEM images (Figure 3a) confirm the crystalline nature of the dark MnO_x_ needles, showing ordered spots or strings that were associated with well-defined planes. The MnO_x_ needles did not have a monocrystalline nature. According to the HRTEM images, they seemed to be formed by the aggregation of very small MnO_x_ nanoparticles; in some cases even subnanometric domains seemed to be observed. The STEM-HAADF image (Figure 3b) shows the presence of bright domains associated with the MnO_x_ needles. However, a more attenuated, homogeneous, and continuous brightness was observed in the sample as a whole. The dispersion of Si and Mn was studied through STEM-HAADF corrected for spherical aberration (Cs). The mapping of the selected elements is included in Figure 4. According to the brightness in the STEM-HAADF images, although Mn was concentrated in the regions associated with the dark needles observed in TEM and HRTEM, the entire sample, including the mesoporous silica counterpart, had significant Mn content. In conclusion, Mn was present in all components of the nanozyme: in the needle-shaped MnO_x_ domains and dispersed in the Mn-UVM-7 type silica matrix.

**Figure 5:**
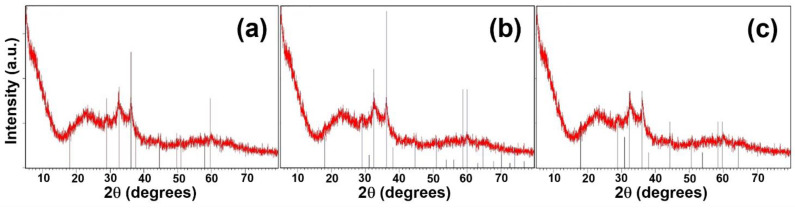
High-angle XRD pattern of the Mn-nanozyme compared to the patterns of (**a**) Mn_2_O_3_ (JCPDS file nº 00-006-0540), (**b**) Mn_3_O_4_ (JCPDS file nº 00-001-1127), and (**c**) MnSiO_3_ (JCPDS file nº 00-012-0181).

The XRD pattern of the material was consistent with electron microscopy observations. In the high-angle XRD domain (Figure 5), we observed a set of low-intensity peaks that could be attributed to various crystalline phases: mainly Mn_2_O_3_ and Mn_3_O_4_, although the presence of MnSiO_3_ was not completely ruled out. Although there was an important coincidence of signals for similar 2θ (°) values, the position and relative intensity of the observed peaks seemed to fit better with the Mn_2_O_3_ oxide (it was probably the dominant MnO_x_ phase). In any case and despite the high Mn content, the low intensity of the signals was related to the small crystallite size of the entities that defined the needles observed by TEM. On the other hand, no signal was detected in the low-angle XRD domain. Again, the small UVM-7 type nanoparticle size of the silica-rich component, with very low numbers of mesopores and particles, excessively limited the number of 2D-unit cells and particles associated with the pore array and excluded the observation of the typical low-angle signal of surfactant-assisted mesoporous silicas.

**Figure 6:**
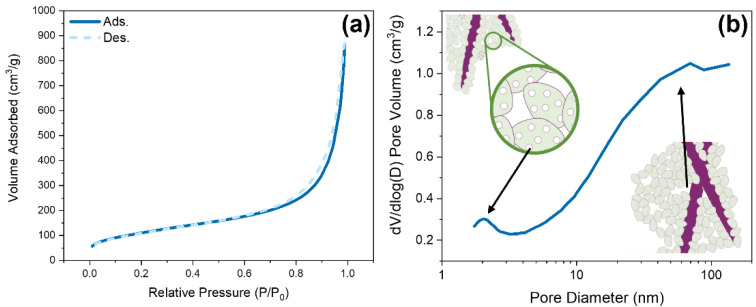
(**a**) N_2_ adsorption–desorption isotherm; (**b**) BJH pore size distribution of the Mn-nanozyme. In the insets are included schematic representations of the intraparticle mesopores and the large interparticle pores.

N_2_ adsorption–desorption isotherms confirmed the presence of hierarchical porosity in the nanozyme (Figure 6a). A slight increase in the adsorption was observed at relative pressures in the range of 0.2 ≤ P/P_0_ ≤ 0.4, followed by a more significant increase that took place, especially at values of P/P_0_ > 0.75. No remarkable hysteresis loops were detected, indicating the absence of cage-like pores. This suggested that there should not be significantly different barriers between adsorption and desorption processes. The bimodal porous character was seen more clearly in the pore size distribution curve (Figure 6b). A low-intensity but very well-defined peak was observed with a maximum at 2.95 nm (according to the application of the BJH model). This pore in the mesopore range was generated by the template effect of the CTAB micelles (after their removal by calcination) and corresponded to the typical mesoporosity in MCM-41 and UVM-7 type silicas, among others. The second peak in the curve showed a great heterogeneity in sizes. It extended over a wide range: between 4 and 100 nm (without ruling out the possibility of larger pores that escaped the detection window of the technique). This textural-type porosity associated with the voids between Mn-UVM-7 silica nanoparticles and MnO_x_ needles implied the existence of pores in the meso- and macroranges. As expected, in our nanozyme the large pores were the dominants, considering the small size of the silica-based nanoparticles. Despite the high Mn content and the nonporous nature of the MnO_x_ needles, the nanozyme showed a high BET area (409 m^2^/g) and a significant BJH pore volume (1.52 cm^3^/g).

**Figure 7:**
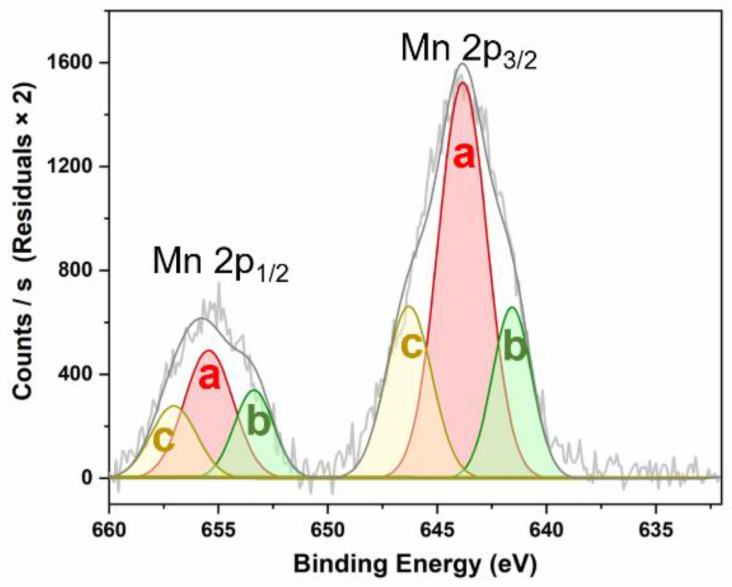
XPS Mn2p spectrum of the Mn-nanozyme. Its deconvolution allowed identifying Mn in different oxidation states: (**a**) Mn^3+^, (**b**) Mn^2+^, and (**c**) Mn^4+^.

From the XRD and TEM data, it is evident that the synthesized Mn nanozyme could contain Mn both in the MnO_x_ needles and in the porous silica phase. Therefore, the presence of Mn in various oxidation states was expected. Then, XPS analysis was used to determine the different oxidation states of Mn present in our sample. For Mn 2p, there were two asymmetric signals associated with Mn 2p_3/2_ and 2p_1/2_ (Figure 7) [66,67]. The spectrum was fitted with a minimum number of six peaks (Figure 7). The most intense peaks at 643.9 and 655.4 eV were assigned to Mn^3+^ 2p_3/s_ and 2p_1/2_, respectively. This was consistent with the presence of Mn_2_O_3_ as the main phase that made up the MnO_x_ needles. The coexistence of other Mn-rich phases, such as Mn_3_O_4_ and even MnSiO_3_ (probably at the MnO_x_-silica interface), was consistent with the detection of some Mn(II) centers. Thus, the peaks at binding energies of 641.7 and 653.2 eV were associated with Mn^2+^ 2p_3/2_ and 2p_1/2_, respectively. Finally, the observation of two additional peaks at 657.1 (Mn^4+^ 2p_3/2_) and 646.3 eV (Mn^4+^ 2p_1/2_) suggested the coexistence of Mn(IV), probably incorporated into the silica Mn-UVM-7, substituting the centers of Si(IV) probably as isolated sites [66,67]. The O1s XPS spectrum showed three typical signals at binding energies of 503.9, 533.1, and 535.4 eV that could be assigned to oxygen atoms in MnO_x_, SiO_2_, and Si-OH species, respectively [68].

### 3.3. Multienzyme-like Activity of Mn-Nanozyme

**Figure 8:**
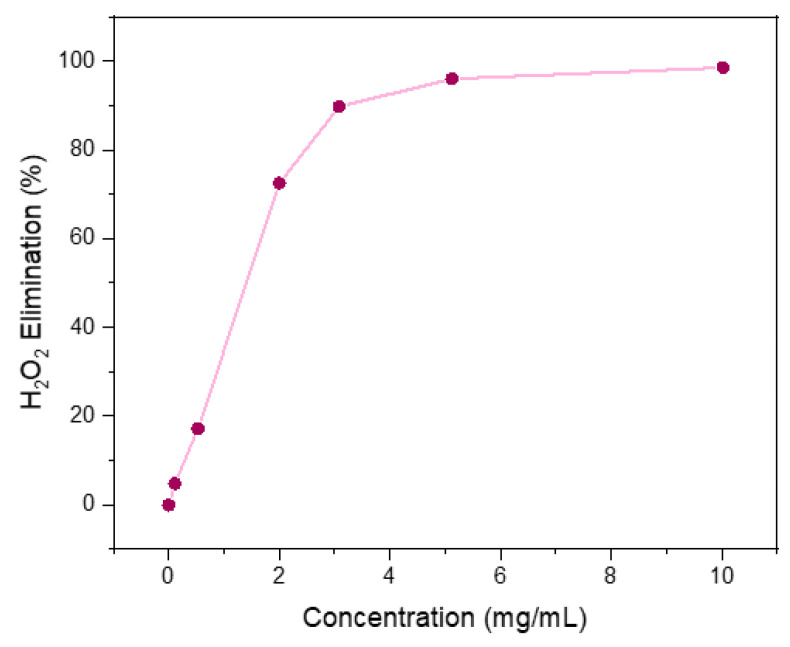
Hydrogen peroxide evolution against the Mn-Nanozyme concentration.

We investigated the catalase activity of the Mn-nanozyme. As expected, the composite exhibited catalase activity in a concentration-dependent manner (Figure 8), and more than 50% of the hydrogen peroxide was decomposed by 2 mg of composite per mL of H_2_O_2_ (1%) in 15 min, with full decomposition observed for 4 mg/mL. This result confirmed that the Mn-nanozyme presented catalase-like activity. However, we found it curious to observe that, apparently, an increase in the initial hydrogen peroxide concentration did not imply a greater degree of progress in the reaction. For this reason, we carried out a kinetic study of the process, designing additional experiences.

**Figure 9:**
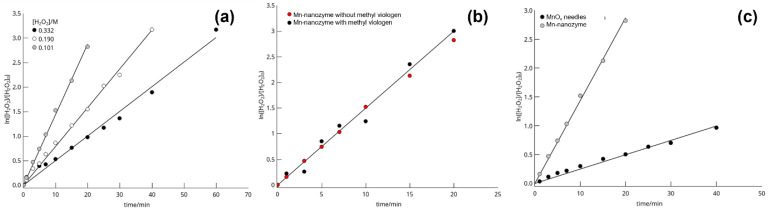
(**a**). ln([H_2_O_2_]/[H_2_O_2_]_0_) vs. time plot for 1mg/mL of Mn-nanozyme and different H_2_O_2_ concentrations; (**b**). ln([H_2_O_2_]/[H_2_O_2_]_0_) vs. time plot for 1mg/mL of Mn-nanozyme, [H_2_O_2_]_0_ = 0.1 M, and [methylviologen] = 0.2 M; (**c**) ln([H_2_O_2_]/[H_2_O_2_]_0_) vs. time plot for [H_2_O_2_]_0_ = 0.1 M, 1 mg/mL of Mn-nanozyme, and 0.57 mg/mL of catalyst without silica.

The complete kinetic study of the catalase activity of the synthesized material is illustrated in Figure 9, which shows the consumption of hydrogen peroxide under various experimental conditions. In all the plots, it was observed that the value of the logarithm of the ratio x = [H_2_O_2_]/[H_2_O_2_]_t=0_ varied linearly with reaction time, implying first-order kinetics with respect to peroxide. The value of the apparent rate constants (k, slope of the lines) calculated from the least-squares regression is given in Table 2, together with the linear correlation coefficient, initial peroxide concentration, and other relevant experimental conditions for the five kinetic runs carried out (denoted as 1–5). Figure 9a shows the dependence of the reaction rate on the initial concentration of hydrogen peroxide. It was observed that the k value decreased with increasing the concentration of the reactant (see experiments 1, 2, and 3 in Table 2). Figure 9b compares the kinetics of the Mn-nanozyme with others carried out under the same conditions, but in the presence of methylviologen, a molecule that is widely used as a hydroxyl radical scavenger [69]. The experiment demonstrated that the addition of an excess of this compound had no appreciable effect on the rate (see k values of experiments 1 (reference) and 5 in Table 2). Finally, Figure 9c compares the peroxide disappearance rate using two catalysts, namely MnO_x_ needles both coated and uncoated with mesoporous silica. For the latter material, the silica coating significantly improved the rate of the catalytic process, as assessed by the k values of experiment 1 (coated), and 4 (uncoated) gathered in Table 2.

**Scheme 1:**
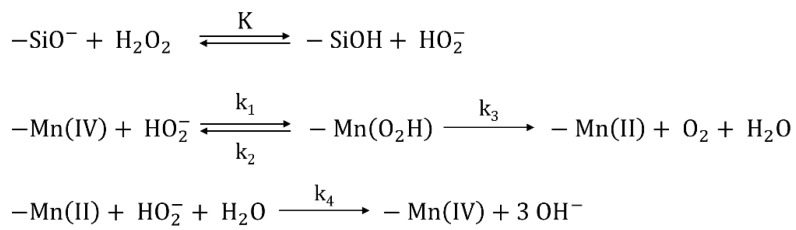
Proposed mechanism for the catalase activity.

The above observations could be explained on the basis of the simplified mechanism shown in Scheme 1. The first step of the mechanism described the acid–base interaction of hydrogen peroxide with the silica coating. This step was responsible for the difference in reaction rates observed for those experiments using bare or silica-coated MnO_x_ needles (Figure 9c, experiments 1 and 4 in Table 2). Thus, if it is admitted that the reaction proceeded through an ionic mechanism, as proposed in Scheme 1, the redox interaction between the Mn centers (electron deficient) and H_2_O_2_ was greatly facilitated when the peroxide was deprotonated on the surface of the silica pores, which resulted in an increase in the ka value for the coated material.

It was further proposed that the Mn oxidation numbers with catalytic activity were mainly (IV) and (II) since they were the most stable and compatible with bielectronic transfers. Nevertheless, steps implying monoelectronic transfers between the IV and III states of the type

−Mn^IV^(HO_2_^−^) → Mn^III^ + HO_2_ (3)

could be proposed, which would be followed by the formation of dioxygen in the liquid phase [70],

HO_2_ + H_2_O_2_ → O_2_ + HO + H_2_O (4)

and finally, OH radicals would restore the (IV) oxidation state of manganese:

−Mn^III^ + OH → Mn^IV^ + OH^−^ (5)

However, if the reaction were to proceed mainly along the path indicated by Equations (3)–(5), the addition of methylviologen should slow down (see experiments 1 and 5 in Table 2) or even inhibit the reaction through OH radical sequestering. However, no appreciable variation was observed between the *k* values measured for experiments 1 and 5. The alternative proposed in Scheme 1, in the third step, is the formation of Mn^II^ to Mn^IV^ centers and its reoxidation by the hydrogen peroxide itself in the basic medium provided by the silica gel pores. The rate law associated with the mechanism in Scheme 1, under the assumption that the SiO^−^ and SiOH centers are in equilibrium and that the active Mn centers reach the steady state, is given by Equation (6):

(6) $$\frac{-d{\left[H_{2}O_{2}\right]}_{T}}{dt}=\frac{2k_{1}k_{3}k_{4}b\left[\mathrm{Mn}\right]{\left[H_{2}O_{2}\right]}_{T}}{\left(k_{1}k_{3}+k_{2}k_{4}+k_{3}k_{4}\right)+k_{3}k_{4}b{\left[H_{2}O_{2}\right]}_{T}},b=\frac{K^{\prime }}{1+K^{\prime }},K^{\prime }=K\frac{\left[-{\mathrm{SiO}}^{-}\right]}{\left[-\mathrm{SiOH}\right]}$$

where [H_2_O_2_]_T_ is the analytical concentration of hydrogen peroxide determined by titration, and the meanings of the constants are as shown in Scheme 1. If it is assumed that the reoxidation of Mn^II^ centers is a slow step, Equation (6) simplifies to Equation (7), which is of the first order with respect to hydrogen peroxide, as observed:

(7) $$\frac{-d{\left[H_{2}O_{2}\right]}_{T}}{dt}=2k_{4}b\left[\mathrm{Mn}\right]{\left[H_{2}O_{2}\right]}_{T}$$

Furthermore, Equation (7) allows the endowment of chemical meaning to the empirical rate coefficients collected in Table 2, as *k* = 2*k*_4_*b*.

Finally, the dependence of the rate constant on the hydrogen peroxide initial concentration (experiments 1–3 in Table 2) can be rationalized by the parameter b appearing in Equation (7). It depends on the equilibrium ratio of the number of deprotonated and protonated silanol groups. It was obvious that, with increasing the initial concentration of peroxide, a Brönsted acid, the ratio and, thus, the kinetic constant decreased.

We concluded that the silica coating of MnO_x_ needles in which Mn appeared in multiple oxidation states, accelerated hydrogen peroxide decomposition efficiently because the silica coating facilitated proton transfer from the substrate, while the various oxidation states of manganese inhibited the radical pathways. Obviously, the term “radical” must include superoxide anions, which could be transformed into dioxygen at the oxide surface through reactions of the following type: Mn^m+^ + O_2_^−^ → Mn^(m−1)+^ + O_2_. The proposed reaction mechanism, consistent with the experimental data, revealed the non-innocent character of silica from a catalytic point of view. Thus, in addition to preventing the aggregation of the MnOx needles, allowing the access of substrates through the hierarchical pore system, it played a key role in providing our nanozyme with catalase activity.

**Figure 10:**
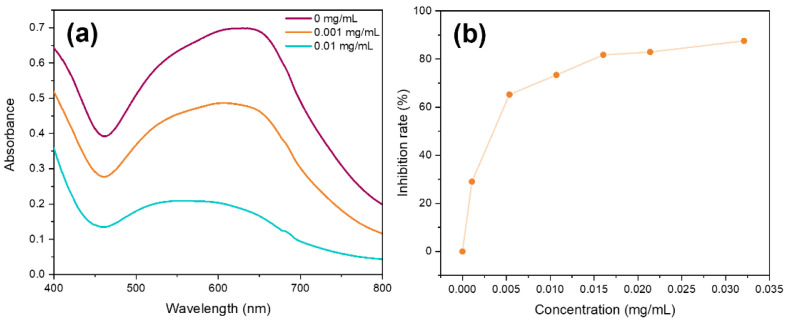
(**a**) UV-visible absorption spectra of different concentrations of Mn-Nanozyme in the superoxide dismutase assay; (**b**) Inhibition rate percentages of superoxide radicals against the concentration of the Mn-Nanozyme.

On the other hand, in the assay for superoxide dismutase activity, the composite was observed to present positive SOD-like activity. This was evident due to the inhibition of the formation of blue-colored formazan (Figure 10a). The percent inhibition of NBT reduction was enhanced by increasing the composite concentration in the reaction medium (Figure 10b). This suggested that the Mn-nanozyme, in a concentration-dependent manner, could scavenge superoxide radicals, avoiding the reduction of NBT to formazan. In this case, the reaction seemed to take place through a radical mechanism typical of a nanozyme containing metals such as Mn (with different accessible oxidation states) and without the participation of mesoporous silica in the reaction mechanism. In the first stage, hydrogen peroxide would be generated through the reaction ˙O_2_^−^ + 2H^+^ → H_2_O_2_ thanks to the oxidation of active manganese centers (Mn^m+^ → Mn^(m+1)+^ + e^−^). In the second stage, oxygen would be generated, ˙O_2_^−^ → O_2_, with a consequent reduction in the Mn centers (Mn^(m+1)+^ + e^−^ → Mn^m+^).

The inhibition rate of the superoxide dismutase activity is usually evaluated by UV-vis measurement of the formation of colored species, such as formazan. The major difference between the methods is based on the way of obtaining the superoxide radicals. In some cases, it is obtained through the photochemical reaction of riboflavin [71] (this work) or with a mixture of xanthine and xanthine oxidase [34,40,41,72].

Although depending on the specific conditions of each experiment, it is sometimes difficult to establish quantitative comparisons, as far as we know, we can affirm that the results obtained with our Mn-nanozyme improve the values described in the bibliography for similar Mn-based catalysts. In a recent article, Pardhiya et al., [71] described the preparation and SOD activity of a nanozyme based on BSA-MnO_2_ nanoparticles. In this case, the inhibition rate achieved was less than 50% for a nanozyme concentration of 300 µg/mL. The values achieved by our nanozyme are significantly better, as we reached a degree of inhibition greater than 50% with a lower nanozyme concentration of only 5 µg/mL. In principle, our nanozyme concentration value (to reach 50% inhibition) is among the lowest previously described for other related Mn materials, which are in the range of 5 to 20 µg/mL [34,40,72]. However, it should be noted that, in our case, the MnO_x_ needles were covered by mesoporous silica nanoparticles, a component that did not participate in SOD activity. Therefore, considering that MnO_x_ was the only component that presented SOD activity and assuming an average compositional formula of MnO_1.33_-SiO_2_, we can estimate that the real concentration of the Mn-nanozyme in our case was significantly lower, around 2.8 µg/mL.

In the case of catalase activity, comparison with other previously published works is much more delicate because the methods used to measure catalytic activity are very different. For example, it can be determined by monitoring the absorbance at 240 nm to calculate the concentration of hydrogen peroxide [72], by the reaction of hydrogen peroxide with a compound to obtain a fluorescent product [40,41,71], or even by determining the O_2_ produced by hydrogen peroxide decomposition [34]. The comparison is also more complex if we consider that the mechanism proposed to explain the catalase activity in our material was bielectronic instead of monoelectronic (as assumed or indicated for other Mn-based nanozymes). Degrees of progress of the reaction of less than 60% after 4 h have been described using very low concentrations of nanozyme. In any case, under our conditions, we reached 90% decomposition in just 20 min using a nanozyme concentration of 1 mg/mL.

Recently the nanozyme definition has been questioned [73]. From a rigorous point of view, it must be a catalyst (in low concentration concerning the substrate) and must follow an identical or very similar mechanism to that of the natural enzyme. In our case, both requirements for the SOD and catalase activity assays are met. In the case of catalase activity, it could be roughly estimated that a concentration of 7.2 × 10^−3^ M in Mn could decompose a solution of up to 0.3 M in H_2_O_2_. Regarding the mechanism that we proposed for catalase activity, it was bielectronic, as in the natural enzyme. However, it differed in how the active center was regenerated, oxidizing Mn(II) to Mn(IV). In addition, from a formal point of view, mathematical Equation (6) could be assimilated into an equation of the Michaelis–Menten type. For this reason, our composite was much closer to a nanozyme than to a nanozyme-like material. On the other hand, in the case of the SOD activity assay, the mechanism was monoelectronic, as in the natural enzyme, and the amounts of catalyst were very low, in the range of a few µg/mL.

## 4. Conclusions

In this work, we described a new nanozyme that presented a very efficient SOD activity, reaching high degrees of inhibition with very low concentrations of catalyst. At the same time, it also showed an effective catalase activity, which translated into quick H_2_O_2_ decomposition in a few minutes. In both cases, and mainly if we refer to the amount of MnO_x_ (the active center), the results improved on the catalytic efficiencies of other related nanozymes described in the literature. Our nanozyme must be considered a composite based on a mixture of manganese oxides and mesoporous silica. The preparative strategy could be viewed as a modification of the original atrane route (one-pot/one-step) in which the processes of hydrolysis and condensation of the atrane complexes of Mn and Si did not start simultaneously (a typical characteristic of the atrane pathway). In this case, the Mn complexes enjoyed a 2 h advantage that allowed the initial formation of MnO_x_ needles. This strategy avoided their agglomeration through the formation of porous silica in the form of aggregated mesoporous nanoparticles. The final nanozyme showed high chemical and morphological homogeneity at the micrometric level. The manganese content was relatively high, at Si/Mn = 1. The Mn was found in different oxidation states (II, III, and IV) and was distributed in nanocrystals that formed micrometric needles, as well as in the silica-rich phase. The synthesis method was highly reproducible, simple to perform, and easily scalable. As previously mentioned, the material showed catalase and SOD activities. While in the case of SOD activity the nanozyme showed a radical mechanism typical of manganese oxide nanoparticles (with monoelectronic transfers), in the case of catalase activity, silica played an important role by facilitating the deprotonation of H_2_O_2_ by acid–base interactions with deprotonated silanol groups, leading to a nonradical mechanism with bielectronic transfers involving Mn(II) and Mn(IV) centers. Moreover, this preparative strategy (one-pot/two-step) could be extended to the syntheses of new nanozymes by combining metal oxide particles (such as CeO_2_, FeO_x_, etc.) and mesoporous silica. The control and modulation of the time took advantage in the hydrolysis and condensation processes of the metals involved with respect to silicon (a very simple parameter to control) and could be the key to the design of this new nanozyme family.

## Figures and Tables

**Table 1:** Selected physical and textural characteristics of the MnO_x_-SiO_2_ composite.

				Mesopore		Large Pore	
Sample	Si/Mn ^1^ Nominal	Si/Mn ^2^ Real	BET area ^3^ (m^2^/g)	Size ^4^ (nm)	Volume ^4^ (cm^3^/g)	Size ^4^ (nm)	Volume ^4^ (cm^3^/g)
Mn nanozyme	6.88	1.01 ± 0.06	405.1	2.95	0.32	26.66	1.20

^1^ Si/Mn nominal molar ratio. ^2^ Si/Mn real molar ratio determined by EDX. ^3^ Surface area was determined by applying the BET model. ^4^ Pore sizes and volumes were determined by applying the BJH model on the adsorption isotherm branches.

**Table 2:** First-order constants calculated from regression lines of Figure 9.

Experiment	[H_2_O_2_]_T_ (M) Initial	*k* × 10^2^ (min^−1^)	R	Observations
1	0.101	1.43 ± 0.02	0.998	Reference reaction
2	0.190	0.79 ± 0.01	0.992	-
3	0.332	0.50 ± 0.01	0.993	-
4	0.101	0.25 ± 0.06	0.991	Uncoated MnO_x_ needles
5	0.101	1.50 ± 0.05	0.991	Reaction in the presence of methylviologen

## Data Availability

Not applicable.
